# Supplementary material for: Rapid evolution of A(H5N1) influenza viruses after intercontinental spread to North America
Source: Nat Commun. 2023 May 29;14:3082. doi: 10.1038/s41467-023-38415-7 (PMC10227026; doi:10.1038/s41467-023-38415-7)
Supplement: Supplementary file 1 — Supplementary information [file 41467_2023_38415_MOESM1_ESM.docx]

Supplementary Materials for

Rapid evolution of A(H5N1) influenza viruses after intercontinental spread to North America

**AUTHOR LIST**: Ahmed Kandeil^1, 2†^, Christopher Patton^1,3†^, Jeremy C. Jones^1†^, Trushar Jeevan^1†^, Walter N. Harrington^1†^, Sanja Trifkovic^1^, Jon P. Seiler^1^, Thomas Fabrizio^1^, Karlie Woodard^1^, Jasmine C. Turner^1^, Jeri-Carol Crumpton^1^, Lance Miller^1^, Adam Rubrum^1^, Jennifer DeBeauchamp^1^, Charles J. Russell^1^, Elena A. Govorkova^1^, Peter Vogel^4^, Mia Kim-Torchetti^5^, Yohannes Berhane^6,7^, David Stallknecht^8^, Rebecca Poulson^8^, Lisa Kercher^1^, Richard J. Webby^1,3^*

**AFFLIATIONS**:

^1^Department of Infectious Diseases, St. Jude Children’s Research Hospital, Memphis, TN 38105, USA.

^2^ Center of Scientific Excellence for Influenza Viruses, National Research Centre, Giza 12622, Egypt.

^3^Department of Microbiology, Immunology, and Biochemistry, University of Tennessee Health Science Center, Memphis, TN 38105, USA.

^4^Comparative Pathology Core, St. Jude Children’s Research Hospital, Memphis, TN 38105, USA.

^5^National Veterinary Services Laboratories, Animal and Plant Health Inspection Service (APHIS), US Department of Agriculture (USDA), Ames, IA 50011, USA.

^6^National Centre for Foreign Animal Disease, Winnipeg, Manitoba R3E 3M4, Canada.

^7^Department of Animal Science, University of Manitoba, Winnipeg, Manitoba R3T 2N2, Canada.

^8^Southeastern Cooperative Wildlife Disease Study, Department of Population Health, College of Veterinary Medicine, The University of Georgia, Athens, GA 30602, USA.

*Corresponding author. Email: richard.webby@stjude.org

^†^These authors contributed equally to this work

**This PDF file includes:**

Supplementary Figs. 1 to 7

Supplementary Tables 1 to 5

**SUPPLEMENTARY FIGURES**


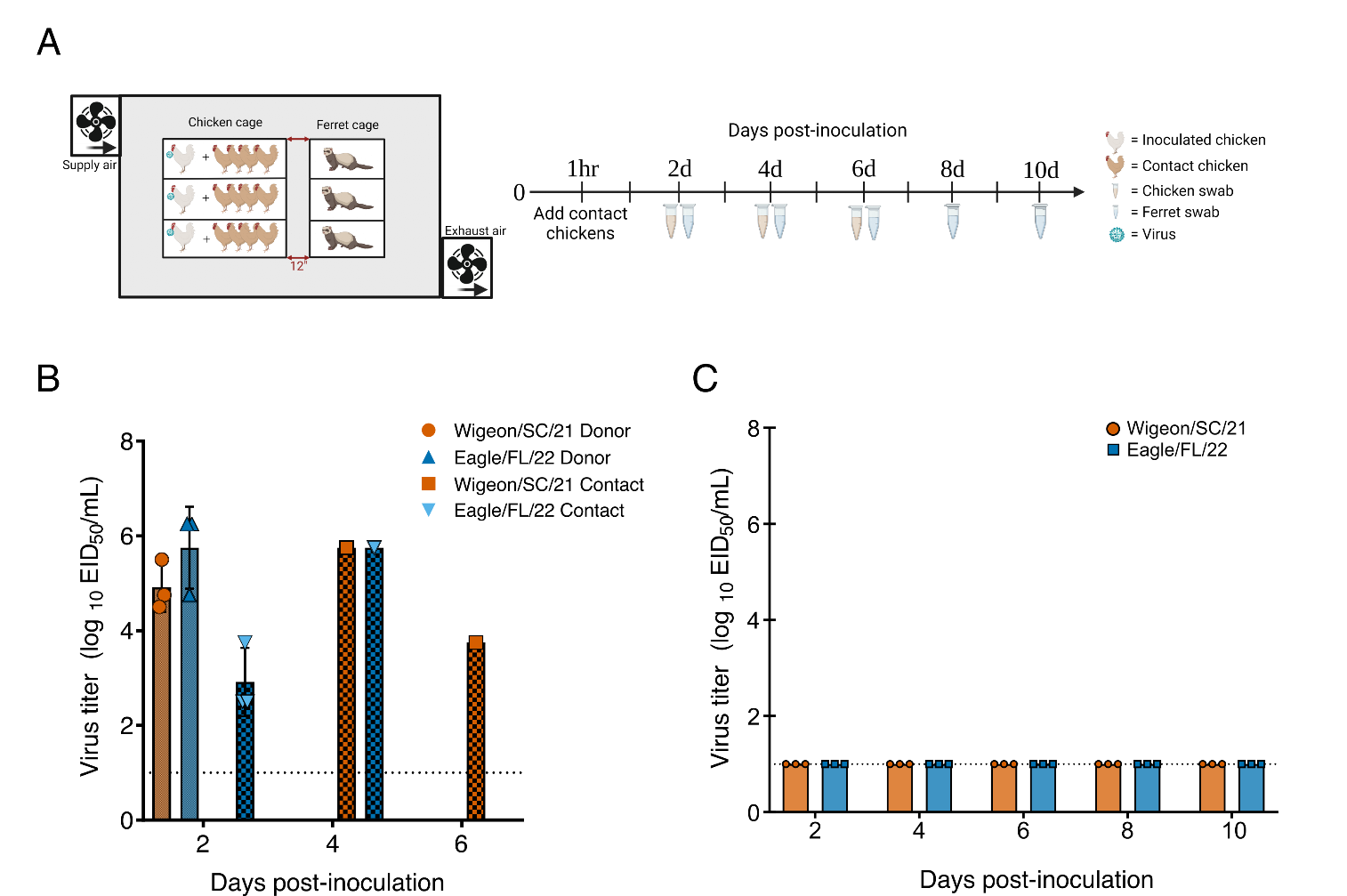


**Supplementary Fig. 1. Replication and transmission of North American HPAI A(H5N1) clade 2.3.4.4b viruses from chickens to ferrets. (A)** Experimental design showing a bioisolator with open caging housing chicken and ferrets at a defined distance. Donor chickens (n = 3 per virus) were inoculated by natural routes (intranasally, intratracheally, intraocularly, intraesophageally) with 10^6^ EID_50_ units of Wigeon/SC/21 or Eagle/FL/22 A(H5N1) virus, then co-housed with 12 naïve contact chickens (n = 4 per donor chicken) at 1 hpi. Swabs were collected at indicated time points. **(B)** Infectious viral titers for cloacal swabs from chickens as determined by EID_50_ assays. Of the 12 contact chickens per virus, 8 remained alive in the Wigeon/SC/21 group, and 9 remained alive in the Eagle/FL/22 group. The values are the mean virus titer (log_10_ EID_50_/mL) ± SD for chickens at 2 dpi. Values at 4 and 6 dpi among the contact chickens represent titers from a single bird that remained positive in each group (Wigeon/SC/21 = 1/11 birds on 4 dpi, and 1/10 birds on 6 dpi; and Eagle/FL/22 = 1/10 birds on 4 dpi, and 0/9 birds on 6 dpi). All donor chickens (solid bars) died by 2 dpi. **(C)** Infectious viral titers in ferret nasal washes. Values are the mean virus titers (log_10_ EID_50_/mL) ± SD. The dashed line indicates the lower limit of virus detection (1.0 log_10_ EID_50_/mL).


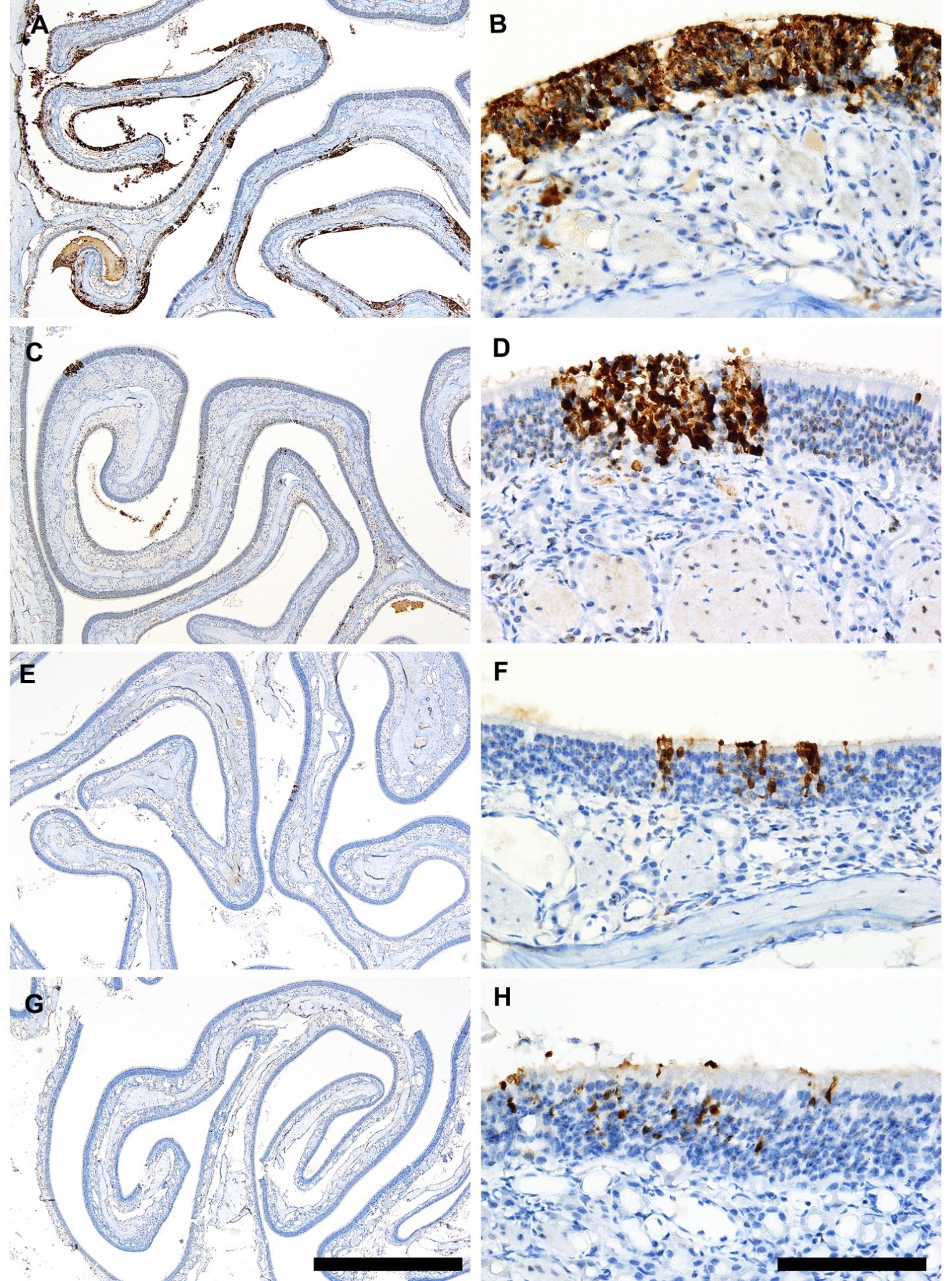


**Supplementary Fig. 2. Upper respiratory tract pathology and viral antigen staining in ferrets inoculated with North American HPAI A(H5N1) clade 2.3.4.4b viruses.** Ferrets were inoculated as described in previous figures with **(A, B)** Scaup/GA/22, **(C, D)** Hawk/NC/22, **(E, F)** Eagle/NC/22, or **(G, H)** Ck/NL/21, and tissues (n =3 ferrets each virus, 5 dpi) were subjected to influenza antigen staining (brown) and pathology evaluation. **(A)** Scaup/GA/22 inoculated ferret turbinates exhibited extensive spread, exfoliation, and degeneration of virus-infected cells, including **(B)** sustentacular cells, olfactory neurons, respiratory epithelium, and occasionally submucosal glands. **(C)** The extent of virus spread in Hawk/NC/22 inoculated ferret tissues was limited to multifocal clusters of neuroepithelial cells, including **(D)** sustentacular cells and olfactory neurons, but only occasional individual ciliated respiratory epithelial cells. **(E)** Eagle/NC/22 inoculated ferrets had very few small foci of virus-positive cells in the olfactory neuroepithelium, and **(F)** virus-infected cells included sustentacular cells and olfactory neurons but no ciliated respiratory epithelial cells. **(G)** In Ck/NL/2 -inoculated ferrets, only two small foci containing virus-infected cells were detected among all three animals, and **(H)** virus infection was limited to sustentacular cells and olfactory neurons; no ciliated respiratory epithelial cells were infected. Results were similar among all three ferrets. Scale bars in A, C, E, and G = 1 mm; those in B, D, F, and H = 100 µm.


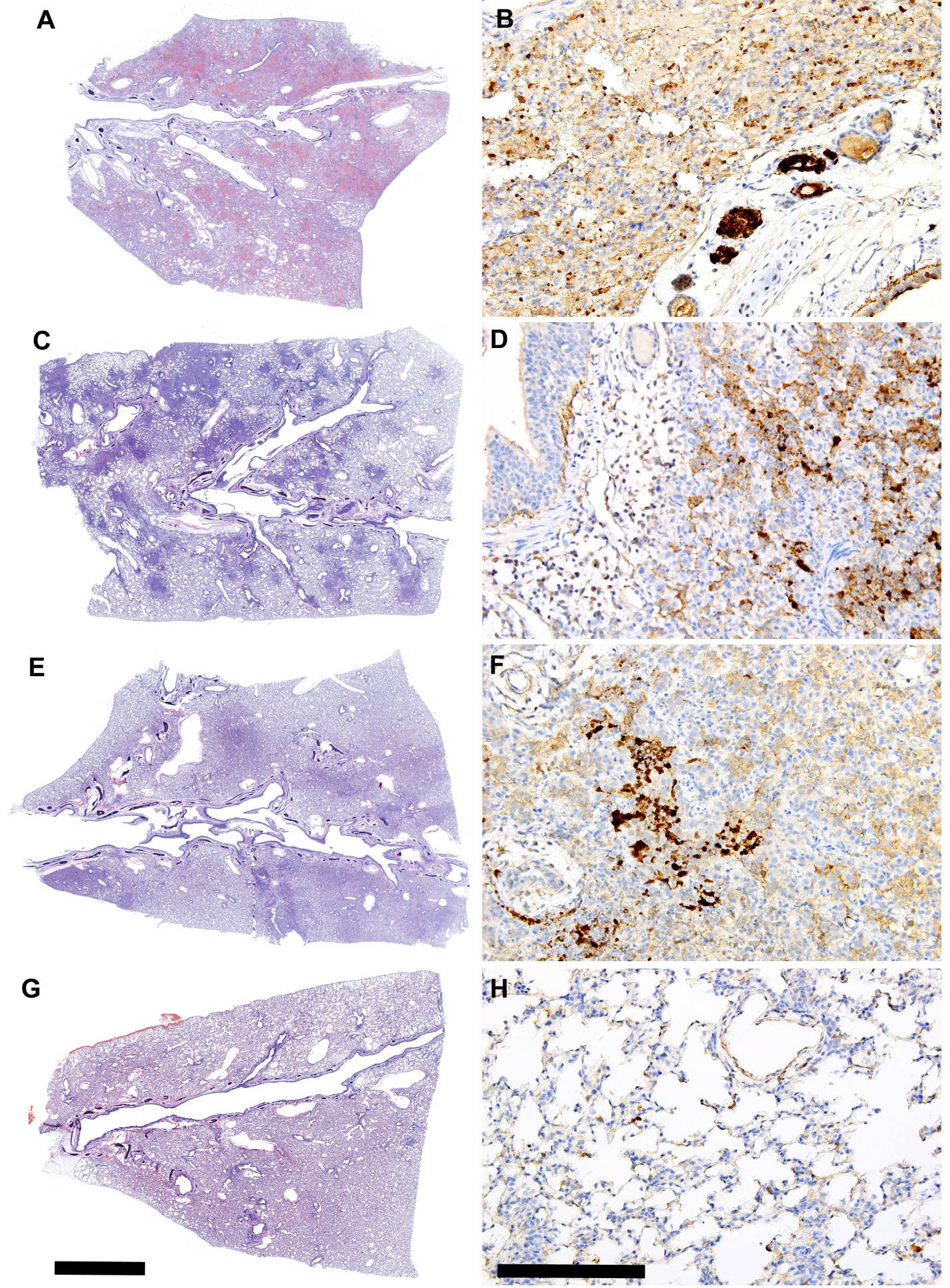
 **Supplementary Fig. 3. Lower respiratory tract pathology and viral antigen staining in ferrets inoculated with North American HPAI A(H5N1) clade 2.3.4.4b viruses.** Ferrets were inoculated as described in previous figures with **(A, B)** Scaup/GA/22, **(C, D)** Hawk/NC/22, **(E, F)** Eagle/NC/22, or **(G, H)** Ck/NL/21, and tissues (n = 3 ferrets per virus, 5 dpi) were subjected to influenza antigen staining (brown) and pathology evaluation. **(A)** Scaup/GA/22 inoculated ferrets exhibited consolidation of entire lung lobes due to extensive necrotizing alveolitis, with associated hemorrhage and edema. Numerous perivascular/peribronchiolar lymphoid nodules were presented in the affected lobes. **(B)** In consolidated alveolar areas, viral antigen was dispersed diffusely, with the scattered apoptotic cell debris and macrophages. **(C)** Hawk/NC/22 inoculated ferrets exhibited lung lesions centered on terminal airways and were generally well-demarcated from the more normal/antigen-negative surrounding parenchyma. **(D)** Viral antigen was mostly concentrated in cellular debris within the bronchial/bronchiolar lumens, but both the bronchiolar epithelium and the submucosal glands were virus negative. **(E)** Eagle/NC/22 inoculated ferret lungs exhibited multifocal areas of increased cellularity characterized by hypertrophic alveolar epithelium. **(F)** Virus-positive debris consisting of neutrophils and cell debris was present in the bronchioles, but the bronchiolar epithelium was negative for virus antigen staining. There was diffuse, mildly positive cytoplasmic labeling of hypertrophic alveolar epithelium, suggestive of a non-cytolytic infection of these cells. **(G)** In Ck/NL/21 inoculated ferret lungs, only one lobe among three ferrets showed any evidence of pulmonary infection. The few lesioned areas were characterized by mildly increased cellularity, but with no detectable cellular exudates or edema. **(H)** In the affected areas, small amounts of virus antigen were detectable on the alveolar surfaces and within a few type I and II pneumocytes, while the bronchiolar epithelium was virus negative and of normal appearance. Results were similar among all three ferrets. Scale bars in A, C, E, and G = 2 mm; those in B, D, F, and H = 200 µm.


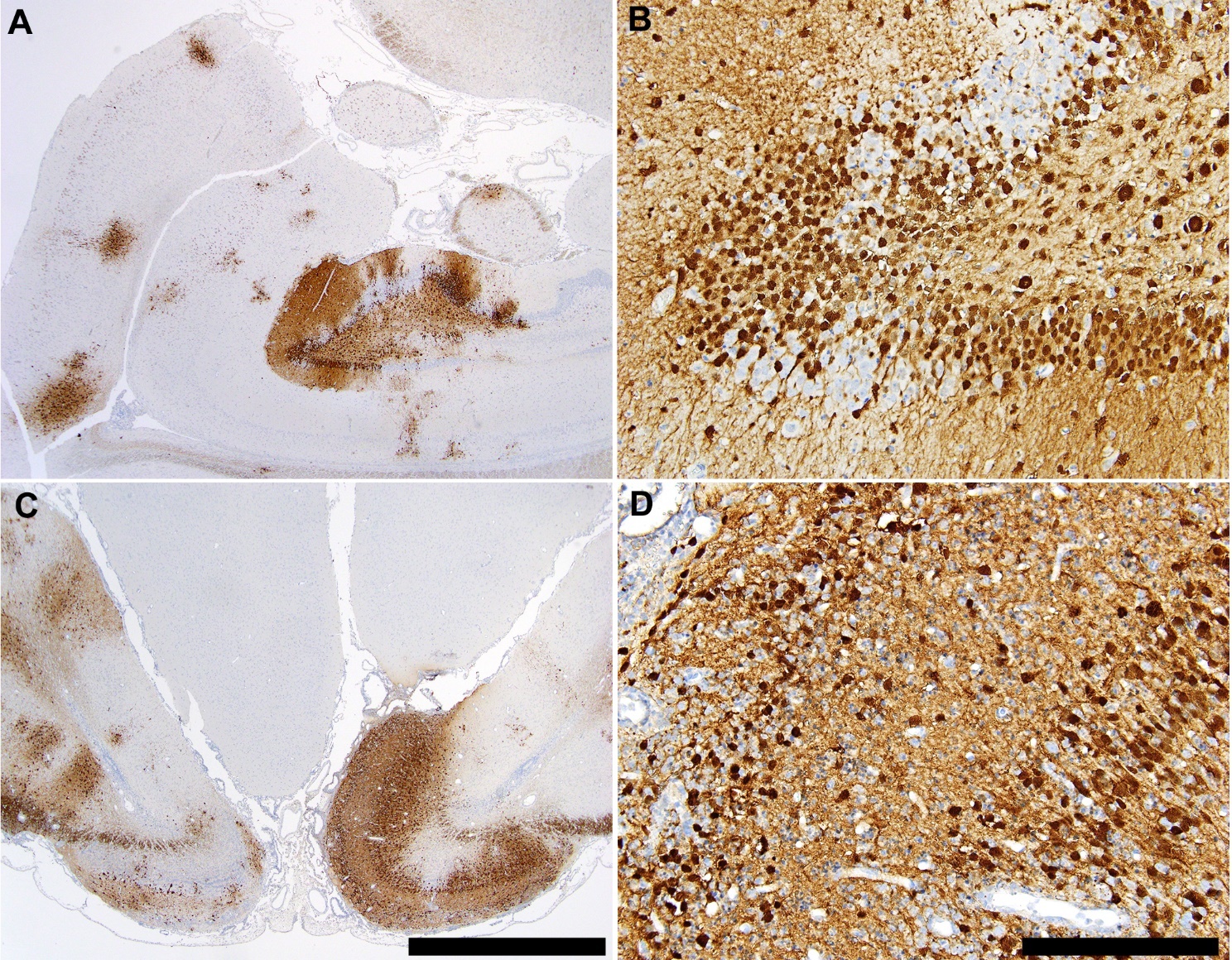


**Supplementary Fig. 4. Pathology and viral antigen staining of the brain in ferrets inoculated with North American HPAI A(H5N1) clade 2.3.4.4b viruses.** Ferrets were inoculated as described in previous figures with **(A, B)** Scaup/GA/22 or **(C, D)** Hawk/NC/22, and tissues (n = 3 ferrets per virus, 5 dpi) were subjected to influenza antigen staining (brown) and pathology evaluation. **(A)** Scaup/GA/22 inoculated ferrets exhibited numerous variably sized foci containing antigen-positive neurons throughout the CNS, which involved the cortex, cerebellum, brainstem, hippocampus, and thalamus, but only a single focus in the olfactory bulb of one ferret. **(B)** A higher-magnification image showing virus antigen in the nuclei and cytoplasm of hippocampal neurons. **(C)** Hawk/NC/22 inoculated ferrets exhibited virus-infected neurons limited to the olfactory bulb and olfactory cortex. **(D)** A higher-magnification image showing virus antigen in the nuclei and cytoplasm of olfactory bulb neurons. Results were similar among all three ferrets. Scale bars in A and C = 2 mm; those in B and D = 200 µm.


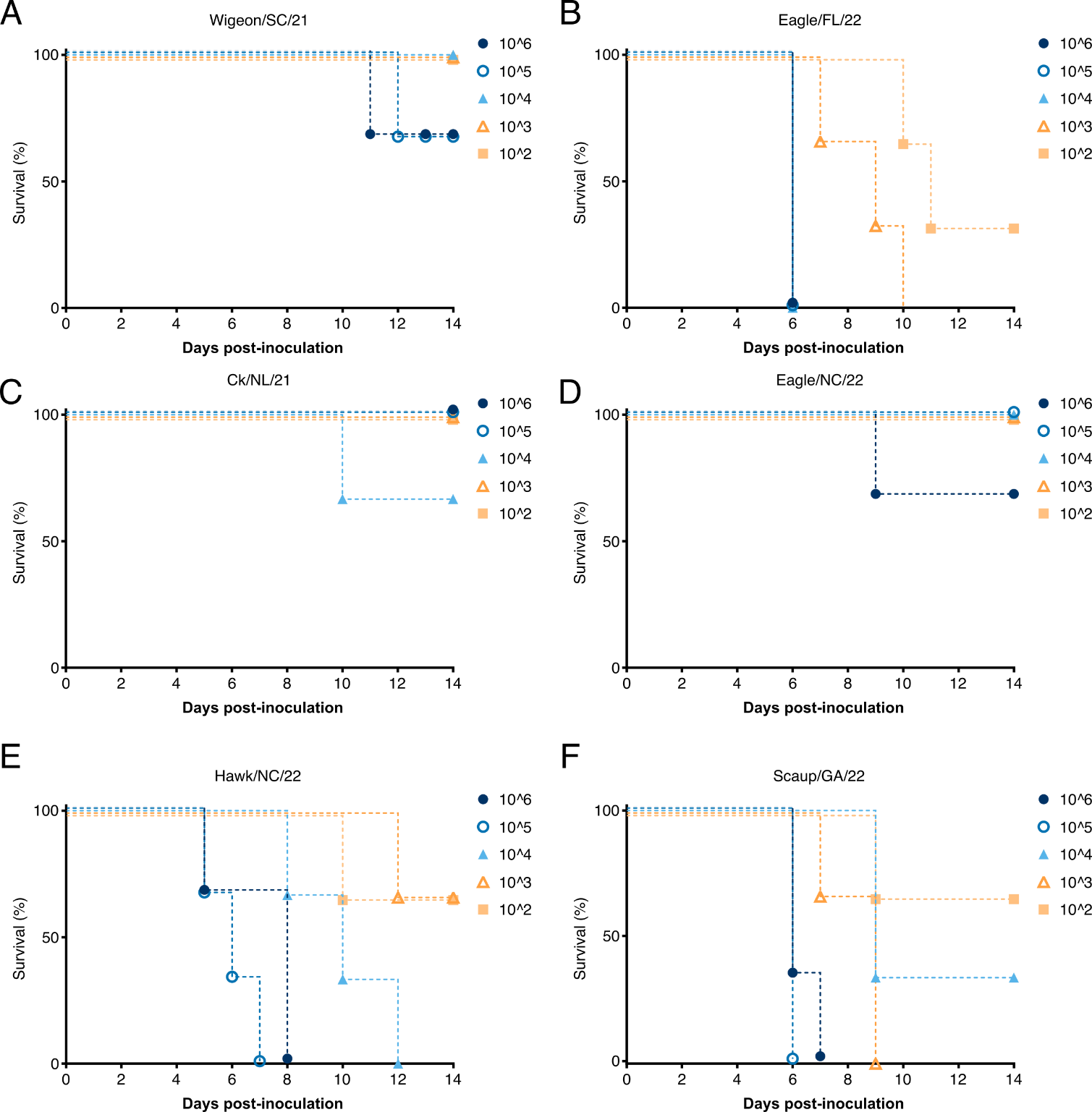


**Supplementary Fig. 5. Survival of mice inoculated with North American HPAI A(H5N1) clade 2.3.4.4b viruses.** BALB/c mice (n = 3 per infectious dose per virus) were inoculated intranasally with the indicated dose (log_10_ TCID_50_/mL) of **(A)** Wigeon/SC/21, **(B)** Eagle/FL/22 **(C)** Ck/NL/21, **(D)** Eagle/NC/22, **(E)** Hawk/NC/22, or **(F)** Scaup/GA/22. Mice were weighed daily and monitored for mortality (humane endpoint or loss of ≥ 25% of their body weight). The Kaplan–Meier method was used to estimate the probability of survival.


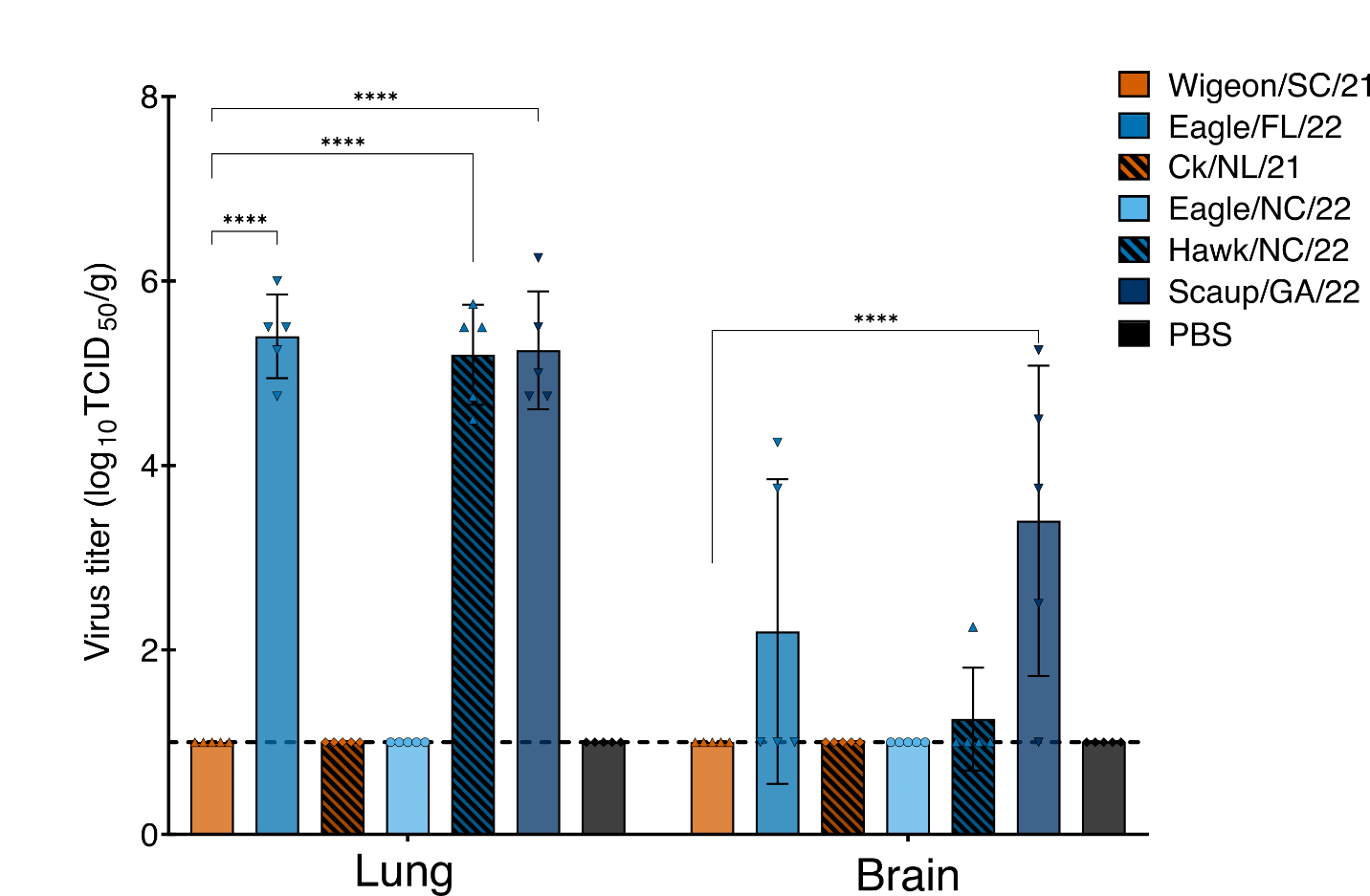


**Supplementary Fig. 6. Replication of North American HPAI A(H5N1) clade 2.3.4.4b viruses in the lungs and brains of mice.** BALB/c mice were intranasally inoculated (10^4^ EID_50_ units) each A(H5N1) influenza virus. Tissues were collected from mice (n = 5 per virus) on 5 dpi, and infectious viral titers were determined in MDCK cells. Values represent the individual animal titers (symbols) and mean virus titers (log_10_ TCID_50_ per gram of wet tissue) ± SD. The dashed line indicates the lower limit of virus detection (1.0 log_10_ TCID_50_ per gram of wet tissue). *P* values were calculated using two-way ANOVA with Tukey’s multiple-comparison post hoc test. ****, *P*< 0.0001.


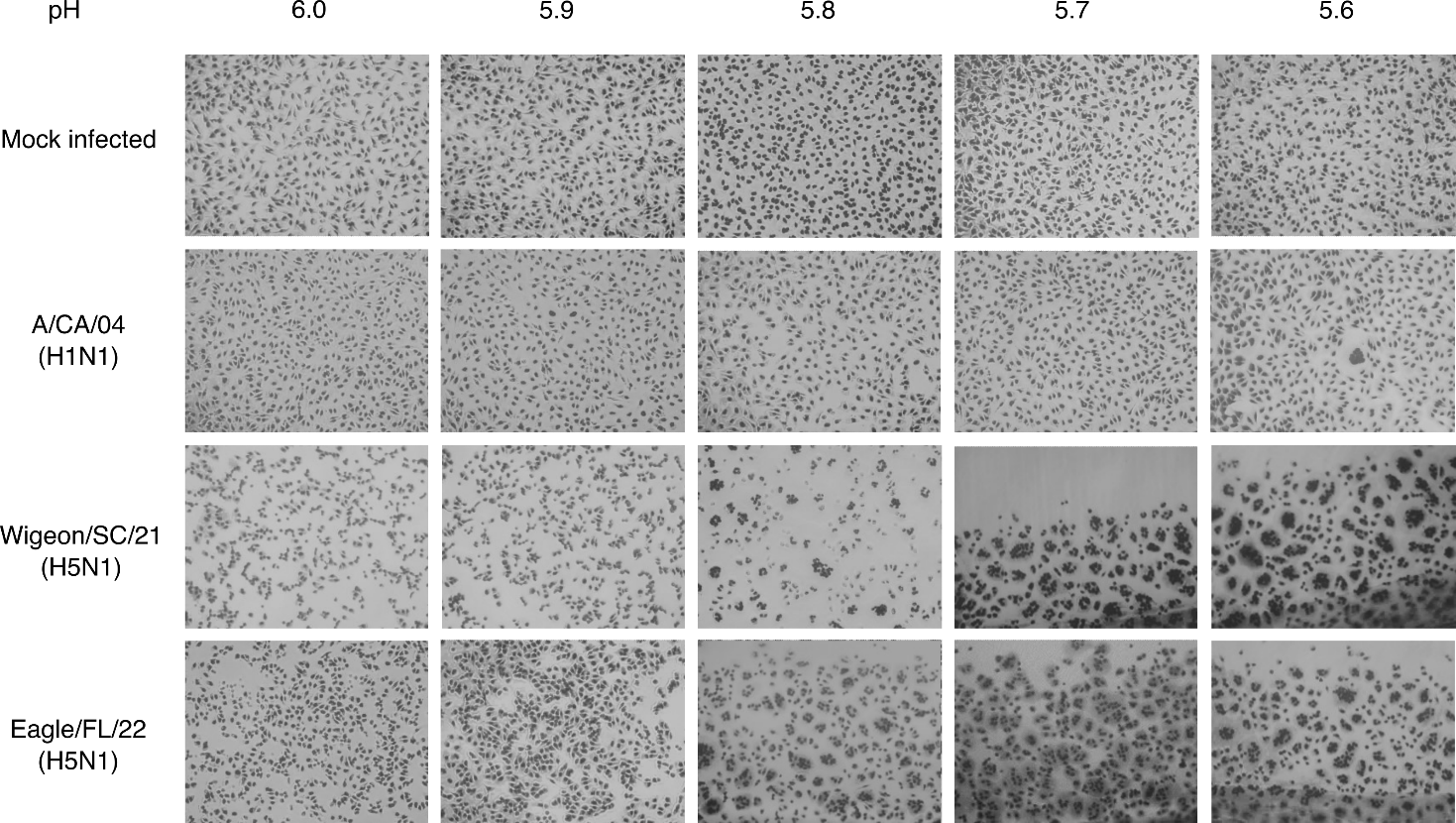


**Supplementary Fig. 7. Syncytium formation in Vero cells inoculated with North American HPAI A(H5N1) clade 2.3.4.4b viruses.** Vero cells were either mock inoculated (PBS) or inoculated with Wigeon/SC/21 A(H5N1), Eagle/FL/22A (H5N1), or A/CA/04 (H1N1)pdm09 virus at an MOI of 3. The HA activation pH was measured by syncytium formation assays in Vero cells; the micrographs are representative results at 6 hpi for the A(H5N1) viruses and at 24 hpi for the A(H1N1)pdm09 virus. Mock inoculated or A(H1N1)pdm09 inoculated vero cells were used as negative and positive controls, respectively. The HA activation pH was defined as the highest pH at which syncytium formation was observed. The resolution was 0.1 pH units. Total magnification was 100X.

**SUPPLEMENTARY TABLES**

**Supplementary Table 1.** Antigenic characterization of North American HPAI A(H5N1) clade 2.3.4.4b viruses

| Influenza A(H5Nx) virus | Subtype | Mean HI titers of post-infection ferret sera against influenza virus^a^ | | |
| --- | --- | --- | --- | --- |
|  |  | A/Fujian-Sanyuan/21099/2017 | A/Astrakhan/3212/2020 | A/Gyrfalcon/WA/  41088-6/2014 |
| Wigeon/SC/21​ | H5N1 | 20 | 80 | 40 |
| Eagle/FL/22​ | H5N1 | 20 | 160 | 80 |
| Ck/NL/21 | H5N1 | 40 | 160 | 160 |
| Eagle/NC/22 | H5N1 | 40 | 160 | 160 |
| Hawk/NC/22​ | H5N1 | 20 | 160 | 80 |
| Scaup/GA/22​ | H5N1 | 20 | 160 | 80 |
| *Reference influenza virus* | | | | |
| A/Fujian-Sanyuan/21099/2017 | H5N6 | **80** | 160 | 160 |
| A/Astrakhan/3212/2020 | H5N8 | 80 | **320** | 320 |
| A/Gyrfalcon/WA/41088-6/2014 | H5N8 | 160 | 320 | **320** |

^a^HI titers are expressed as the reciprocal values (e.g., 10 versus 1:10) of the highest dilution of serum that inhibited 4 HA units of virus. The results are the arithmetic mean titer of positive sera (HI titer >10). The titers of serum samples with homologous viruses are shown in bold and are underlined.

| **Supplementary Table 2.** GenBank accession numbers of North American HPAI A(H5N1) clade 2.3.4.4b viruses used in the study | | | | | | | | | |  |
| --- | --- | --- | --- | --- | --- | --- | --- | --- | --- | --- |
|  |  |  |  |  |  |  |  |  |  |  |
| Influenza A(H5N1) virus | GenBank accession number for gene segment: | | | | | | | |  |  |
|  | HA | NA | MP | NP | NS | PA | PB1 | PB2 |  |  |
| A/Lesser scaup/Maryland/LC-EESC-024/2022 | OP499861 | OP499862 | OP499863 | OP499865 | OP499864 | OP499860 | OP499859 | OP499858 |  |  |
| A/Bald eagle/Florida/W22-114/2022 | OP470804 | OP470805 | OP470806 | OP470807 | OP470808 | OP470809 | OP470810 | OP470811 |  |  |
| A/Red-shouldered hawk/North Carolina/W22-121/2022 | OQ442195 | OQ442196 | OQ442197 | OQ442198 | OQ442199 | OQ442200 | OQ442201 | OQ442202 |  |  |
| A/Lesser scaup/Florida/W22-129A/2022 | OP470812 | OP470813 | OP470814 | OP470815 | OP470816 | OP470817 | OP470818 | OP470819 |  |  |
| A/Bald eagle/Florida/W22-134-OP/2022 | OP377436 | OP377437 | OP377438 | OP377439 | OP377440 | OP377441 | OP377442 | OP377443 |  |  |
| A/Bald eagle/North Carolina/W22-140/2022 | OP377622 | OP377623 | OP377624 | OP377625 | OP377626 | OP377627 | OP377628 | OP377629 |  |  |
| A/Bald eagle/Florida/W22-142/2022 | OP470797 | OP470798 | OP470799 | OP470800 | OP470801 | N/D | OP470802 | OP470803 |  |  |
| A/Lesser scaup/Georgia/W22-143/2022 | OP470765 | OP470766 | OP470767 | OP470768 | OP470769 | OP470770 | OP470771 | OP470772 |  |  |
| A/Lesser scaup/Georgia/W22-145A/2022 | OP470773 | OP470774 | OP470775 | OP470776 | OP470777 | OP470778 | OP470779 | OP470780 |  |  |
| A/Lesser scaup/Georgia/W22-145B/2022 | OP470749 | OP470750 | OP470751 | OP470752 | OP470753 | OP470754 | OP470755 | OP470756 |  |  |
| A/Lesser scaup/Georgia/W22-145C/2022 | OP470716 | OP470717 | OP470718 | OP470719 | OP470720 | OP470721 | OP470722 | OP470723 |  |  |
| A/Lesser scaup/Georgia/W22-145D/2022 | OP470757 | OP470758 | OP470759 | OP470760 | OP470761 | OP470762 | OP470763 | OP470764 |  |  |
| A/Lesser scaup/Georgia/W22-145E/2022 | OP470781 | OP470782 | OP470783 | OP470784 | OP470785 | OP470786 | OP470787 | OP470788 |  |  |
| A/Bald eagle/Florida/W22-153A/2022 | OP377534 | OP377535 | OP377536 | OP377537 | OP377538 | OP377539 | OP377540 | OP377541 |  |  |
| A/Bald eagle/Florida/W22-153B/2022 | OP377461 | OP377462 | OP377463 | OP377464 | OP377465 | OP377466 | OP435198 | OP377467 |  |  |
| A/Hooded merganser/Florida/W22-154/2022 | OP377630 | OP377631 | OP377632 | OP377633 | OP377634 | OP377635 | OP377636 | OP377637 |  |  |
| A/Great blue heron/Florida/W22-160/2022 | OP377542 | OP377543 | OP377544 | OP377545 | OP377546 | OP377547 | OP377548 | OP377549 |  |  |
| A/Black vulture/Florida/W22-161/2022 | OP377486 | OP377487 | OP377488 | OP377489 | OP377490 | OP377491 | OP377492 | OP377493 |  |  |
| A/Gull/Florida/W22-162/2022 | OP377420 | OP377421 | OP377422 | OP377423 | OP377424 | OP377425 | OP377426 | OP377427 |  |  |
| A/Great-horned owl/Florida/W22-163A/2022 | OP377510 | OP377511 | OP377512 | OP377513 | OP377514 | OP377515 | OP377516 | OP377517 |  |  |
| A/Great-horned owl/Florida/W22-163C/2022 | OP377638 | OP377639 | OP377640 | OP377641 | OP377642 | OP377643 | OP377644 | OP377645 |  |  |
| A/Brown pelican/North Carolina/W22-164/2022 | OP377606 | OP377607 | OP377608 | OP377609 | OP377610 | OP377611 | OP377612 | OP377613 |  |  |
| A/Black vulture/Florida/W22-167/2022 | OP377574 | OP377575 | OP377576 | OP377577 | OP377578 | OP377579 | OP377580 | OP377581 |  |  |
| A/Black vulture/Florida/W22-168/2022 | OP377453 | OP377454 | OP377455 | OP377456 | OP377457 | OP377458 | OP377459 | OP377460 |  |  |
| A/Ring-billed gull/Florida/W22-169/2022 | OP377372 | OP377373 | OP377374 | OP377375 | OP377376 | OP377377 | OP377378 | OP377379 |  |  |
| A/Snow goose/Kansas/W22-174B/2022 | OP377590 | OP377591 | OP377592 | OP377593 | OP377594 | OP377595 | OP377596 | OP377597 |  |  |
| A/Snow goose/Kansas/W22-177B/2022 | OP377428 | OP377429 | OP377430 | OP377431 | OP377432 | OP377433 | OP377434 | OP377435 |  |  |
| A/Bald eagle/Kansas/W22-185/2022 | OP377646 | OP377647 | OP377648 | OP377649 | OP377650 | OP377651 | OP377652 | OP377653 |  |  |
| A/Bald eagle/North Carolina/W22-186/2022 | OP377404 | OP377405 | OP377406 | OP377407 | OP377408 | OP377409 | OP377410 | OP377411 |  |  |
| A/Bald eagle/North Carolina/W22-229/2022 | OP377445 | OP377446 | OP377447 | OP377448 | OP377449 | OP377450 | OP377451 | OP377452 |  |  |
| A/Royal tern/Florida/W22-245A/2022 | OP377614 | OP377615 | OP377616 | OP377617 | OP377618 | OP377619 | OP377620 | OP377621 |  |  |
| A/Royal tern/Florida/W22-245B/2022 | OP377412 | OP377413 | OP377414 | OP377415 | OP377416 | OP377417 | OP377418 | OP377419 |  |  |
| A/Snow goose/Kansas/W22-260/2022 | OP377518 | OP377519 | OP377520 | OP377521 | OP377522 | OP377523 | OP377524 | OP377525 |  |  |
| A/Bald eagle/Florida/W22-189/2022 | OP221398 | OP221400 | OP221399 | OP221401 | OP221402 | OP221403 | OP222197 | OP221404 |  |  |
| A/Bald eagle/Georgia/W22-194A/2022 | OP221382 | OP221383 | OP221384 | OP221385 | OP221386 | OP221387 | OP221388 | OP221389 |  |  |
| A/Bald eagle/Georgia/W22-194B/2022 | OP221301 | OP221302 | OP221303 | OP221304 | OP221305 | OP221306 | OP221307 | OP221308 |  |  |
| A/Bald eagle/Florida/W22-195/2022 | OP221327 | OP221328 | OP221329 | OP221330 | OP221331 | OP221332 | OP221333 | OP221334 |  |  |
| A/Bald eagle/Kansas/W22-197/2022 | OP221041 | OP221042 | OP221043 | OP221044 | OP221045 | OP221046 | OP221047 | OP221048 |  |  |
| A/Red-tailed hawk/Kansas/W22-198/2022 | OP499866 | OP499867 | OP499868 | OP499869 | OP499870 | OP499871 | OP499872 | OP499873 |  |  |
| A/Snow goose/Kansas/W22-199A/2022 | OP221350 | OP221351 | OP221352 | OP221353 | OP221354 | OP221355 | OP221356 | OP221357 |  |  |
| A/Snow goose/Kansas/W22-199B/2022 | OP221358 | OP221359 | OP221360 | OP221361 | OP221362 | OP221363 | OP221364 | OP221365 |  |  |
| A/Snow goose/Kansas/W22-199C/2022 | OP221390 | OP221391 | OP221392 | OP221393 | OP221394 | OP221395 | OP221396 | OP221397 |  |  |
| A/snow goose/Kansas/W22-199D/2022 | OP221285 | OP221286 | OP221287 | OP221288 | OP221289 | OP221290 | OP221291 | OP221292 |  |  |
| A/Snow goose/Kansas/W22-199E/2022 | OP221374 | OP221375 | OP221376 | OP221377 | OP221378 | OP221379 | OP221380 | OP221381 |  |  |
| A/Snow goose/Kansas/W22-199F/2022 | OP221293 | OP221294 | OP221295 | OP221296 | OP221297 | OP221298 | OP221299 | OP221300 |  |  |
| A/Bald eagle/Georgia/W22-202/2022 | OP221335 | OP221336 | OP221337 | OP221338 | OP221339 | OP221340 | OP221341 | OP221342 |  |  |
| A/Bald eagle/South Carolina/W22-205/2022 | OP221366 | OP221367 | OP221368 | OP221369 | OP221370 | OP221371 | OP221372 | OP221373 |  |  |
| A/Lesser snow goose/North Dakota/ND-04/2022 | OP377330 | OP377331 | OP377332 | OP377333 | OP377334 | OP377335 | OP377336 | OP377337 |  |  |
| A/Lesser snow goose/North Dakota/ND-05/2022 | OP377364 | OP377365 | OP377366 | OP377367 | OP377368 | OP377369 | OP377370 | OP377371 |  |  |
| A/Lesser snow goose/North Dakota/ND-06/2022 | OP377322 | OP377323 | OP377324 | OP377325 | OP377326 | OP377327 | OP377328 | OP377329 |  |  |
| A/Ross' goose/North Dakota/ND-08/2022 | OP377566 | OP377567 | OP377568 | OP377569 | OP377570 | OP377571 | OP377572 | OP377573 |  |  |
| A/Bald eagle/Virginia/W22-306/2022 | OP377380 | OP377381 | OP377382 | OP377383 | OP377384 | OP377385 | OP377386 | OP377387 |  |  |
| A/Muscovy duck/Florida/W22-316/2022 | OP377396 | OP377397 | OP377398 | OP377399 | OP377400 | OP377401 | OP377402 | OP377403 |  |  |
|  |  |  |  |  |  |  |  |  |  |  |
| N/D, not done. |  |  |  |  |  |  |  |  |  |  |

| **Supplementary Table 3.** GISAID accession numbers of North American HPAI A(H5N1) clade 2.3.4.4b viruses used in this study | | | | | | |
| --- | --- | --- | --- | --- | --- | --- |
|  |  |  |  |  |  |  |
| Influenza A(H5N1) virus | GISAID accession number for gene segment: | | Collected (Y-M-D) | Originating laboratory | Submitting laboratory | Authors |
|  | HA | PB2 |  |  |  |  |
| A/Anas platyrhynchos/Belgium/8777-003/2021 | EPI1966308 | EPI1966305 | 2021-06-27 | Sciensano - Animal Infectious Diseases, Belgium | Sciensano, Department of Animal Infectious Diseases, Belgium | Van Borm, S.; Roupie, V.; Lambrecht, B.; Mathijs, E.; Steensels, M. |
| A/Chroicocephalus ridibundus/Belgium/13464/2020 | EPI1942890 | EPI1942887 | 2021-12-09 | Sciensano - Animal Infectious Diseases, Belgium | Sciensano, Department of Animal Infectious Diseases, Belgium | Van Borm, S.; Vandenbussche, F.; Roupie, V.; Lambrecht, B.; Steensels, M. |
| A/Cinnamon teal/Utah/AH0166335/2021 | EPI1933057 | EPI1933054 | 2021-08-06 | Import from public domain | Import from public domain | USDA National Veterinary Services Laboratories; USDA Wildlife Services; Utah Veterinary Diagnostic Laboratory; Franzen,K.M.; Love,E.R.; Chinh,T.T.; Killian,M.L.; Koster,L.G.; Bevins,S.N.; Linder,T.J.; Lenoch,J.B.; Dilione,K. |
| A/Duck/Bangladesh/17D1738/2021 | EPI1986144 | EPI1986141 | 2021-08-19 | International Centre for Diarrhoeal Disease Research, Bangladesh | International Centre for Diarrhoeal Disease Research, Bangladesh | Hossain, M.E.; Miah, M.; Alam, S.; Hasan, R.; Davis, C.T.; Mott, A.J.; Davis, W.; Chowdhury, S.; Rahman, M.Z. |
| A/Duck/Bangladesh/17D1815/2021 | EPI1997134 | EPI1997131 | 2021-12-05 | International Centre for Diarrhoeal Disease Research, Bangladesh | International Centre for Diarrhoeal Disease Research, Bangladesh | Hossain, M.E.; Miah, M.; Alam, S.; Hasan, R.; Davis, C.T.; Mott, A.J.; Davis, W.; Chowdhury, S.; Rahman, M.Z |
| A/Duck/Bangladesh/18D1795/2021 | EPI1997142 | EPI1997139 | 2021-12-10 | International Centre for Diarrhoeal Disease Research, Bangladesh | International Centre for Diarrhoeal Disease Research, Bangladesh | Hossain, M.E.; Miah, M.; Alam, S.; Hasan, R.; Davis, C.T.; Mott, A.J.; Davis, W.; Chowdhury, S.; Rahman, M.Z |
| A/Duck/Bangladesh/19D1742/2021 | EPI1986199 | EPI1986196 | 2021-09-01 | International Centre for Diarrhoeal Disease Research, Bangladesh | International Centre for Diarrhoeal Disease Research, Bangladesh | Hossain, M.E.; Miah, M.; Alam, S.; Hasan, R.; Davis, C.T.; Mott, A.J.; Davis, W.; Chowdhury, S.; Rahman, M.Z |
| A/Duck/Bangladesh/19D1818/2021 | EPI1997262 | EPI1997259 | 2021-12-22 | International Centre for Diarrhoeal Disease Research, Bangladesh | International Centre for Diarrhoeal Disease Research, Bangladesh | Hossain, M.E.; Miah, M.; Alam, S.; Hasan, R.; Davis, C.T.; Mott, A.J.; Davis, W.; Chowdhury, S.; Rahman, M.Z |
| A/Duck/Czech Republic/18520-2/2021 | EPI1921688 | EPI1921685 | 2021-09-27 | State Veterinary Institute Prague, Czeck Republic | State Veterinary Institute Prague, Czech Republic | Nagy, A.; Cernikova, L.; Stara, M. |
| A/Gallus gallus/Belgium/11372-0001/2021 | EPI1966300 | EPI1966297 | 2021-08-31 | Sciensano - Animal Infectious Diseases, Belgium | Sciensano, Department of Animal Infectious Diseases, Belgium | Van Borm, S.; Roupie, V.; Lambrecht, B.; Mathijs, E.; Steensels, M. |
| A/Gallus gallus/Belgium/16070-003/2021 | EPI1946078 | EPI1946075 | 2021-12-07 | Sciensano - Animal Infectious Diseases, Belgium | Sciensano - Animal Infectious Diseases, Belgium | Van Borm, S.; Roupie, V.; Lambrecht, B.; Mathijs, E.; Steensels, M. |
| A/Golden eagle/Finland/9378-21VIR7689-12/2021 | EPI1945461 | EPI1945458 | 2021-07-01 | Finnish Food Authority, Finland | Istituto Zooprofilattico Sperimentale Delle Venezie, Italy | Tammiranta, N.; Kantala, T.; Laamanen, I.; Gadd, T.; Zecchin, B.; Fusaro, A.; Schivo, A.; Salviato, A.; Palumbo, E.; Milani, A.; Giussani, E.; Pastori, A.; Monne, I.; Terregino, C. |
| A/Goose/Tyumen/33-53V/2021 | EPI1922997 | EPI1922994 | 2021-10-07 | FBUZ Center of Hygiene and Epidemiology in Tyumen oblast, Russian Federation | State Research Center of Virology and Biotechnology (VECTOR), Russian Federation | Goncharova, N.; Susloparov, I.; Kolosova, N.; Danilenko, A.; Bulanovich, J.; Marchenko, V.; Ryzhikov, A. |
| A/Grey heron/Czech Republic/25338-2/2021 | EPI2024067 | EPI2024064 | 2021-12-18 | State Veterinary Institute Prague | State Veterinary Institute Prague | Nagy, A.; Stara, M.; Cernikova, L. |
| A/Greylag goose/Netherlands/21037809-001/2021 | EPI1929971 | EPI1929974 | 2021-10-31 | Wageningen Bioveterinary Research, Netherlands | Wageningen Bioveterinary Research, Netherlands | Beerens, N.; Harders, F.; Pritz-Verschuren, S.; Roose, M.; Germeraad, E.; Engelsma, M.; Heutink, R. |
| A/Greylag goose/Sweden/SVA211103SZ0398/FB004410/M-2021 | EPI1928247 | EPI1928244 | 2021-11-01 | National Veterinary Institute, Sweden | National Veterinary Institute, Sweden | Not available |
| A/Mallard/Alaska/AH0176804/2021 | EPI1933012 | EPI1932163 | 2021-07-30 | Import from public domain | Import from public domain | USDA Wildlife Services; USDA National Veterinary Services Laboratories; Washington Animal Disease Diagnostic Laboratory; Chinh,T.; Love,E.; Franzen,K.; Killian,M.; Koster,L.; Bevins,S.; Dilione,K.; Lenoch,J.; Linder,T. |
| A/Mallard/Alaska/AH0176840/2021 | EPI1932147 | EPI1932145 | 2021-07-30 | Import from public domain | Import from public domain | USDA Wildlife Services; USDA National Veterinary Services Laboratories; Washington Animal Disease Diagnostic Laboratory; Chinh,T.; Love,E.; Franzen,K.; Killian,M.; Koster,L.; Bevins,S.; Dilione,K.; Lenoch,J.; Linder,T. |
| A/Mallard/Italy/21VIR6957-6/2021 | EPI1947271 | EPI1947268 | 2021-08-20 | Istituto Zooprofilattico Sperimentale Delle Venezie, Italy | Istituto Zooprofilattico Sperimentale Delle Venezie, Italy | Milani, A.; Fusaro, A.; Schivo, A.; Salviato, A.; Palumbo, E.;Zecchin, B.; Giussani, E.; Pastori, A.; Monne, I.; Terregino, C. |
| A/Mallard/Netherlands/12/2021 | EPI1963344 | EPI1963341 | 2021-12-23 | Erasmus Medical Center, Netherlands | Erasmus Medical Center, Netherlands | Not available |
| A/Mallard/New York/AH0179244/2021 | EPI1932236 | EPI1932232 | 2021-08-11 | Import from public domain | Import from public domain | USDA National Veterinary Services Laboratories; USDA Wildlife Services; Cornell Diagnostic Laboratory; Franzen,K.M.; Love,E.R.; Chinh,T.T.; Killian,M.L.; Koster,L.G.; Bevins,S.N.; Linder,T.J.; Lenoch,J.B.; Dilione,K. |
| A/Mallard/New York/AH0179250/2021 | EPI1932242 | EPI1932239 | 2021-08-11 | Import from public domain | Import from public domain | USDA National Veterinary Services Laboratories; USDA Wildlife Services; Cornell Diagnostic Laboratory; Franzen,K.M.; Love,E.R.; Chinh,T.T.; Killian,M.L.; Koster,L.G.; Bevins,S.N.; Linder,T.J.; Lenoch,J.B.; Dilione,K. |
| A/Mallard/Utah/AH0152319/2021 | EPI1933064 | EPI1933061 | 2021-08-03 | Import from public domain | Import from public domain | USDA National Veterinary Services Laboratories; USDA Wildlife Services; Cornell Diagnostic Laboratory; Franzen,K.M.; Love,E.R.; Chinh,T.T.; Killian,M.L.; Koster,L.G.; Bevins,S.N.; Linder,T.J.; Lenoch,J.B.; Dilione,K. |
| A/Swan/Romania/16905-22VIR2749-1/2021 | EPI2014985 | EPI2014982 | 2021-12-08 | Istituto Zooprofilattico Sperimentale delle Venezie, EU/OIE/Reference Laboratory and FAO Reference Centre for Avian Influenza and Newcastle Disease, Italy | Istituto Zooprofilattico Sperimentale delle Venezie, Italy | Barbuceanu, F.; Onita, I.; Neicut, A.; Motiu, R.; Burlacu, R.; Barbierato, G.; Zecchin, B.; Fusaro, A.; Schivo, A.; Salviato, A.; Palumbo, E.; Giussani, E.; Monne, I.; Terregino, C. |
| A/Turkey/Italy/21VIR9117-2/2021 | EPI1947341 | EPI1947338 | 2021-11-04 | Istituto Zooprofilattico Sperimentale Delle Venezie, Italy | Istituto Zooprofilattico Sperimentale Delle Venezie, Italy | Milani, A.; Fusaro, A.; Schivo, A.; Salviato, A.; Palumbo, E.;Zecchin, B.; Giussani, E.; Pastori, A.; Monne, I.; Terregino, C. |

**Supplementary Table 4.**Pathogenicity, clinical disease, transmission, and serology of North American HPAI A(H5N1) clade 2.3.4.4b viruses in ferrets

| Influenza A(H5N1) virus^a^ | Donor ferrets^a^ | | | | | | | Contact ferrets^g^ | |
| --- | --- | --- | --- | --- | --- | --- | --- | --- | --- |
|  | No. surviving/ | Weight loss^b^ | Temperature increase^b^ | Respiratory signs^c^ | Neurologic signs^d^ | Relative inactivity index^e^ | Range of post infection HI titers^f^ | Nasal Wash Titer^g^ | Range of post exposure HI titers^f^ |
| Wigeon/SC/21 | 3/3 | 1/3 (17 ± 9.6) | 3/3 (1.2 ± 0.5) | 0/3 | 0/3 | 0.1 | 160 − 640 | - | - |
| Eagle/FL/22 | 0/3 | 3/3 (20 ± 6.9) | 3/3 (3.3 ± 0.5) | 3/3 | 3/3 | 1.7 | NT | - | - |
| Ck/NL/21 | 3/3 | 0/3 (13 ± 2.7) | 3/3 (0.9 ± 0.1) | 0/3 | 0/3 | 0 | 10 − 40 | - | - |
| Eagle/NC/22 | 1/3 | 3/3 (15 ± 6.1) | 3/3 (3.0 ± 1.1) | 0/3 | 2/3 | 0.8 | 320 | - | - |
| Hawk/NC/22 | 0/3 | 3/3 (16 ± 10.5) | 3/3 (2.0 ± 0.7) | 2/3 | 3/3 | 1.3 | NT | - | - |
| Scaup/GA/22 | 0/3 | 3/3 (18 ± 2.3) | 3/3 (1.8 ± 0.9) | 3/3 | 3/3 | 1 | NT | - | - |

^a^For each virus, groups of three donor ferrets were lightly anesthetized with isoflurane and inoculated intranasally with 10^6^ EID_50_ units of virus in 1.0 mL PBS.

^b^Maximum change in mean weight (percentage) or temperature (°C) ± SD is shown in parentheses among donor ferrets.

^c^Respiratory signs were sneezing, wheezing, nasal discharge, and labored breathing among donor ferrets.

^d^Neurologic signs among donor ferrets were hind-limb paresis, ataxia, torticollis, and tremors.

^e^Determined by observation for 14 days. Assessment of the activity level was done based on the following scoring system: 0, alert and playful; 1, alert but playful only when stimulated; 2, alert but not playful when stimulated; 3, neither alert nor playful when stimulated. The relative inactivity index before virus inoculation was 0^1^.

^f^Hemagglutination inhibition titers against homologous viruses 24 days after inoculation (expressed as reciprocal values, e.g., 10 versus 1:10).

^g^For each virus, groups of three naïve contact ferrets were placed directly into the same cage of the inoculated donors 24 hr post-donor inoculation. Contact animal nasal washes were collected in the same manner as donor animals. No contact animals shed detectable virus at any time point.

NT, not tested (all ferrets in the group were dead)

(-) indicates values were below the assay limits of detection (TCID_50_ assay – 1 log_10_ TCID_50_/mL; HI assay – 1:10 sera dilution)

**Supplementary Table 5.**Primer sequences used to generate reverse genetics viruses.

| Influenza gene | Primer orientation and nucleotide position (5' - 3') | Primer sequence |
| --- | --- | --- |
| PA | Bm^a^-PA-1 | TATTCGTCTCAGGGAGCGAAAGCAGGTAC |
|  | Bm-PA-2233R | ATATCGTCTCGTATTAGTAGAAACAAGGTACTT |
| PB1 | Bm-PB1-1 | TATTCGTCTCAGGGAGCGAAAGCAGGCA |
|  | Bm-PB1-2341R | ATATCGTCTCGTATTAGTAGAAACAAGGCATTT |
| PB2 | Ba-PB2-1 | TATTGGTCTCAGGGAGCGAAAGCAGGTC |
|  | Ba-PB2-2341R | ATATGGTCTCGTATTAGTAGAAACAAGGTCGTTT |
| NA | Ba-NA-1 | TATTGGTCTCAGGGAGCAAAAGCAGGAGT |
|  | Ba-NA-1413R | ATATGGTCTCGTATTAGTAGAAACAAGGAGTTTTTT |
| NP | Bm-NP-1 | TATTCGTCTCAGGGAGCAAAAGCAGGGTA |
|  | Bm-NP1565R | ATATCGTCTCGTATTAGTAGAAACAAGGGTATTTTTC |

^a^indicates primer introduction of restriction site BsmBI (Bm) or BsaI (Ba)

(R) indicates reverse primer

**SUPPLEMENTARY REFERENCES**

1 Reuman, P. D., Keely, S. & Schiff, G. M. Assessment of signs of influenza illness in the ferret model. *J Virol Methods* **24**, 27-34, doi:10.1016/0166-0934(89)90004-9 (1989).
