# Supplementary material for: Rapid evolution of A(H5N1) influenza viruses after intercontinental spread to North America
Source: Nat Commun. 2023 May 29;14:3082. doi: 10.1038/s41467-023-38415-7 (PMC10227026; doi:10.1038/s41467-023-38415-7)
Supplement: Supplementary file 3 — Reporting Summary [file 41467_2023_38415_MOESM3_ESM.pdf]

nature portfolio

Corresponding author(s): Richard J. Webby

Last updated by author(s): Apr 25, 2023

Reporting Summary

Nature Portfolio wishes to improve the reproducibility of the work that we publish. This form provides structure for consistency and transparency in reporting. For further information on Nature Portfolio policies, see our Editorial Policies and the Editorial Policy Checklist.

Statistics

For all statistical analyses, confirm that the following items are present in the figure legend, table legend, main text, or Methods section.

n/a	Confirmed	
☐	☒	The exact sample size (n) for each experimental group/condition, given as a discrete number and unit of measurement
☐	☒	A statement on whether measurements were taken from distinct samples or whether the same sample was measured repeatedly
☐	☒	The statistical test(s) used AND whether they are one- or two-sided<br>*Only common tests should be described solely by name; describe more complex techniques in the Methods section.*
☒	☐	A description of all covariates tested
☒	☐	A description of any assumptions or corrections, such as tests of normality and adjustment for multiple comparisons
☐	☒	A full description of the statistical parameters including central tendency (e.g. means) or other basic estimates (e.g. regression coefficient) AND variation (e.g. standard deviation) or associated estimates of uncertainty (e.g. confidence intervals)
☐	☒	For null hypothesis testing, the test statistic (e.g. F, t, r) with confidence intervals, effect sizes, degrees of freedom and P value noted<br>*Give P values as exact values whenever suitable.*
☒	☐	For Bayesian analysis, information on the choice of priors and Markov chain Monte Carlo settings
☒	☐	For hierarchical and complex designs, identification of the appropriate level for tests and full reporting of outcomes
☒	☐	Estimates of effect sizes (e.g. Cohen's d, Pearson's r), indicating how they were calculated

Our web collection on statistics for biologists contains articles on many of the points above.

Software and code

Policy information about availability of computer code

Data collection	Illumina MiSeq Control Software v2.6
Data analysis	CLC Genomics Workbench v22.0.1 (Qiagen); BioEdit v7.2.5 (open source); MEGA v7 (open source); CD-HIT v4.8.1; Augur v15.0.1; TreeTime v0.8.6; Auspice v2.29.1; Prism v9 (GraphPad); BioRender

For manuscripts utilizing custom algorithms or software that are central to the research but not yet described in published literature, software must be made available to editors and reviewers. We strongly encourage code deposition in a community repository (e.g. GitHub). See the Nature Portfolio guidelines for submitting code & software for further information.

Data

Policy information about availability of data

All manuscripts must include a data availability statement. This statement should provide the following information, where applicable:

- Accession codes, unique identifiers, or web links for publicly available datasets
- A description of any restrictions on data availability
- For clinical datasets or third party data, please ensure that the statement adheres to our policy

Data generated in this study are provided in the main manuscript, supplementary information, and/or source data files. GenBank and GISAID sequence accession numbers are provided in supplemental tables 2 and 3.

Research involving human participants, their data, or biological material

Policy information about studies with human participants or human data. See also policy information about sex, gender (identity/presentation), and sexual orientation and race, ethnicity and racism.

Reporting on sex and gender	Human subjects were not used in this study
Reporting on race, ethnicity, or other socially relevant groupings	Human subjects were not used in this study
Population characteristics	Human subjects were not used in this study
Recruitment	Human subjects were not used in this study
Ethics oversight	Human subjects were not used in this study

Note that full information on the approval of the study protocol must also be provided in the manuscript.

Field-specific reporting

Please select the one below that is the best fit for your research. If you are not sure, read the appropriate sections before making your selection.

[x] Life sciences [ ] Behavioural & social sciences [ ] Ecological, evolutionary & environmental sciences

For a reference copy of the document with all sections, see nature.com/documents/nr-reporting-summary-flat.pdf

Life sciences study design

All studies must disclose on these points even when the disclosure is negative.

Sample size	Experimental replicates and multiple independent experiments are detailed in the methods section and figure legends. Pre-study statistical testing for animal sample size/no. was not performed; however, study size and replicate animal nos. conforms to literature practices regarding testing of pathogenicity and transmission in the respective models. (Belser et al., mBio, 13(4), 2022; Bosco-Louth et al., Virology, 496, 2016, pp161-166)
Data exclusions	No data were excluded.
Replication	All experimental replicates were performed according to well described standard operating procedures, and the protocols were utilized uniformly among all investigators in this study. Experimental replicates are listed in detail in the manuscript file. Repeat experiments were consistent and representative of the data presented in each figure or table.
Randomization	Randomization was not performed for in vitro experiments. For in vivo experiments, research animals were randomly assigned to experimental groups/caging systems by animal care staff.
Blinding	Blinding was not performed. This study utilized highly pathogenic influenza viruses, which are USDA select agents housed in animal biosafety level 3+ containment facilities. Best practices and institutional biosafety guidelines require the investigators to be aware of the identity of the virus and/or virus associated samples they are using, as well as the elevated risk characteristics associated with such samples.

Reporting for specific materials, systems and methods

We require information from authors about some types of materials, experimental systems and methods used in many studies. Here, indicate whether each material, system or method listed is relevant to your study. If you are not sure if a list item applies to your research, read the appropriate section before selecting a response.

Materials & experimental systems

n/a	Involved in the study	
[ ]	[x]	Antibodies
[ ]	[x]	Eukaryotic cell lines
[x]	[ ]	Palaeontology and archaeology
[ ]	[x]	Animals and other organisms
[x]	[ ]	Clinical data
[x]	[ ]	Dual use research of concern
[x]	[ ]	Plants

Methods

n/a	Involved in the study	
[x]	[ ]	ChIP-seq
[x]	[ ]	Flow cytometry
[x]	[ ]	MRI-based neuroimaging

Antibodies

Antibodies used	Goat anti-influenza A virus (A/USSR/1977 [H1N1]) (US Biological Life Sciences, # I7650-05E), Donkey anti-goat biotinylated secondary antibody (US Biological Life Sciences, #I1904-28B).
Validation	From the vendor: Recognizes Influenza A, USSR. Reacts with purified virions. Does not react with Influenza B, RSV, Para 1-3 or Adeno. Does not react with HEp-2 cells. May react with chicken cellular proteins.<br>El-shesheny, R., et al. 2018. Sci Reports. 8(1). 10693. doi:10.1038/s41598-018-29079-1. PMID 29691394

Eukaryotic cell lines

Policy information about cell lines and Sex and Gender in Research

Cell line source(s)	Madin–Darby canine kidney (MDCK) cells (ATCC CCL-34), African green monkey kidney (Vero) cells (ATCC CCL-81), Human embryonic kidney (HEK293T) cells (ATCC CRL-3519), Human airway epithelial (Calu-3) cells (ATCC HTB-55), Primary differentiated human airway cultures (MatTek, AIR-100)
Authentication	All cell lines were accompanied by lot-specific certificates of analysis documenting cell type, clone, lot, passage, morphology, species determination (interspecies isoenzyme assay), viability, and growth properties.
Mycoplasma contamination	All cell lines were accompanied by documentation confirming absence of mycoplasma (Hoeschst DNA staining, agar culture), and were subjected to routine testing by ATCC Universal Mycoplasma testing kit (30-1012K) during laboratory culture. All lines remained mycoplasma-negative.
Commonly misidentified lines<br>(See ICLAC register)	No commonly misidentified cell lines were used in this study.

Animals and other research organisms

Policy information about studies involving animals; ARRIVE guidelines recommended for reporting animal research, and Sex and Gender in Research

Laboratory animals	Ferrets (male, 4-6 mo. age); Mice (BALB/c, female, 6-8 wk. age); Chickens (White Leghorn, 6 wk. age, mixed sex)
Wild animals	Wild animals were not used in this study.
Reporting on sex	It is unlikely that findings will differ between sexes of age and approximately weight-matched individuals. Female mice were used to conform with and facilitate comparisons to initial risk assessments of similar clade viruses (Pulit-Penaloza et al., JVI, 89(20), PMID: 26223637) as well as many similar studies including the initial study of this subtype in mice (Lu et al, JVI, 73(7), pp. 5903-5911, PMID: 10364342). Chickens were received from vendor as mixed-sex. Male ferrets were used for animal welfare concerns: the estrus cycle of female ferrets, if prolonged, leads to aplastic anemia and adds undue stress and potential health complications for the research animals.
Field-collected samples	In vitro and in vivo experimentation was performed with egg or cell propagated stocks of influenza virus and did not directly use any field-collected samples.
Ethics oversight	All animal studies were approved by the St. Jude Children's Research Hospital Institutional Animal Care and Use Committee (IACUC, protocol #428) in accordance with the guidelines established by the Institute of Laboratory Animal Resources, approved by the Governing Board of the US National Research Council, and carried out by trained personnel working in a United States Department of Agriculture (USDA)-inspected Animal Biosafety Level 3+ animal facility in accordance with all regulations established by the Division of Agricultural Select Agents and Toxins (DASAT) at the USDA Animal and Plant Health Inspection Service (APHIS), as governed by the United States Federal Select Agent Program (FSAP) regulations (7 CFR Part 331, 9 CFR Part 121.3, 42 CFR Part 73.3).

Note that full information on the approval of the study protocol must also be provided in the manuscript.
